# More than the eyes can see: The worrying scenario of canine leishmaniasis in the Brazilian side of the triple border

**DOI:** 10.1371/journal.pone.0189182

**Published:** 2017-12-12

**Authors:** Vanete Thomaz Soccol, Aline Kuhn Sbruzzi Pasquali, Eliane Maria Pozzolo, André de Souza Leandro, Luciana Chiyo, Rafael Antunes Baggio, Mario Sergio Michaliszyn, Carlos Silva, Patrícia Hoerner Cubas, Ricardo Peterlle, Otacilio Lopes de Souza Paz, Ivana Lucia Belmonte, Alceu Bisetto-Junior

**Affiliations:** 1 UFPR—Graduate Program in Bioprocess Engineering and Biotechnology, Federal University of Paraná, Rua Francisco H dos Santos, Centro Politécnico, Curitiba, Paraná, Brazil; 2 SESA- Secretary of Health of the State of Paraná, Curitiba, Paraná, Brazil and Ninth Health Region, Foz do Iguaçu, Paraná, Brazil; 3 Zoonosis Control Center—CCZ, Foz do Iguaçu Paraná, Brazil; 4 Graduate Program in Environmental Engineering, Positive University, Curitiba, Paraná, Brazil; 5 Vigilância Sanitária, Santa Terezinha de Itaipu, Paraná, Brazil; 6 UFPR- Departamento de Saúde Comunitária, Curitiba, Paraná, Brazil; 7 Laboratório de Análise de Padrões Espaciais e Cartografia Temática (LAPE-CT), Laboratório Pedagógico de Geografia (LABOGEO), Universidade Federal do Paraná, Curitiba, Paraná, Brazil; University of Ostrava, CZECH REPUBLIC

## Abstract

A cross-sectional epidemiological study in the extreme-west of the state of Paraná was carried out to access the prevalence, distribution, and risk variables of canine Visceral Leishmaniases (cVL). This study was conducted in three areas, two cities of far west of Parana state: Foz do Iguaçu (FI) and Santa Terezinha de Itaipu (STI), and along two transects between these two municipalities. To sample the entire urban area, the cities (FI and STI) were divided into a grid of squares of 400 m^2^ (patch). Among the 526 patches, 123 in FI, 40 in the transects and 33 in STI were selected according to the ‘worst scenario’ criterion. In the transect areas, in each 0.86 km five dogs from houses were surveyed to leishmaniasis. In each patch, blood of five dogs from houses (and from neighborhood when necessary) in the areas that seemed to be the most appropriate for the proliferation of vector were surveyed. The infection of the dogs by cVL were assessed using two serological tests were used (cELISA and TR-DPP®), and, for those seropositive for both methods, the PCR method were used. Moreover, dogs presenting clinical signs or cutaneous lesions were sampled to PCR. The identification of *Leishmania* species was confirmed using PCR-RFLP followed by DNA sequencing. Micro, meso and macro scale environmental variables were also surveyed and statistically analyzed. The prevalence rate *Leishmania infantum* was 23.8% in FI, 4.7% in STI and 9.1% in the transects areas. Among the extrinsic variables analysed, the number of vectors and the presence of infected dogs in neighbouring were positively correlated with the occurrence of infected dogs. Dog size was positively correlated with cVL infection, while the quality of the dog’s nutrition affected cVL negatively. As for cutaneous leishmaniasis (CL), the first registry of dogs infected with *L*. *braziliensis* in the region shows that there is potential for transmission in peri-urban areas, since environmental conditions allow the proliferation of vectors capable of transmitting this species of parasite. cVL is widely spread in FI, with high prevalence. This supports the hypothesis that the parasite has been present in the region for longer than previously believed, despite the fact that the presence of leishmaniais in the region has only been recognized recently. It is important to control the population of dogs infected with *L*. *infantum* (parasite and non-antibodies) to prevent the spread of the disease to other dogs and also to people in the region.

## Introduction

Visceral Leishmaniasis (VL) is a zoonotic disease caused by *Leishmania donovani* in the Old and by *L*. *infantum* in the Old and New World. Worldwide, domestic dogs are the primary reservoir of the disease, which is transmitted by the females of some species of sand flies (Phlebotominae) [1].

In the Brazil, from the 1950s to the 1980s, visceral leishmaniasis was only prevalent in disadvantaged rural areas of the Northeast. Since then, the disease has gradually expanded to peri-urban and urban areas, following the population migrations to these areas. From the 1990s, the disease has expanded fast to the Brazilian Southeast [2–7]. In the South region, LV is more recent. The first autochthonous cVL (canine Visceral Leishmaniasis) cases in this region were reported in 2009 in São Borja, state of Rio Grande do Sul [8]. In the state of Santa Catarina, the first autochthonous cases of cVL were reported in 2010, in the city of Florianopolis [9], and in 2013 in the western region (São Miguel do Oeste and Descanso), [10]. Concomitantly, VL has expanded its reach to neighbouring countries (Argentina and Paraguay), where a gradual increase in human deaths due to it has been observed. Strong evidence supports the hypothesis that VL has expanded into urban areas in the south portion of South America [11–13].

The state of Paraná, in southern Brazil, was considered free of VL transmission until 2012, since neither cVL nor *Lu*. *longipalpis* had been reported there [14,15]. Vector of *Leishmania*, *Lu*. *longipalpis*, was sampled for the first time in Foz do Iguaçu in 2012, when the city was declared an area of risk to the disease. Later that year the first autochthonous cases of cVL were reported. Finally, in 2015, the first case of VL was documented, and *L*. *infantum* was confirmed as the aetiological agent [16–19].

The spatial distribution of a pathogenic agent at an area is important to monitor and to control the disease it causes. Since cVL in dogs usually precedes human cases, we used dogs as proxy to assess the spatial distribution and the risk factors for VL in the far west of the state of Paraná. To accomplish this, we conducted a cross-sectional epidemiological study in this area to determine the prevalence, distribution, and risk factors of cVL. To this end, three sampling sites were selected: Foz do Iguaçu (FI), a city adjacent to the triple frontier (Brazil, Argentina and Paraguay); Santa Terezinha do Itaipu (STI), to assess if the parasite has dispersed to nearby cities, and two transects (T1 and T2) between them, to assess if the disease is present or emergent, and how is the dissemination of cVL in this rural area.

## Materials and methods

### Area studied

The city of Foz do Iguaçu (FI, state of Paraná, southern of Brazil, 25°32’49” S, 54°35’17” W) is located in average 192 m above sea level [20]. For this study, the city of Foz do Iguaçu was divided into four areas (A–West; B–East; C—North; and D—South), consistent with the areas delimited by the Health Service. Area A has 64,864 habitants and 7,724 dogs, area B has 93,020 habitants and 16,146 dogs, area C has 123,128 habitants and 23,583 dogs, and area D has 44,120 habitants and 7,530 dogs. The data from dogs was obtained at Zoonosis Control Center, CCZ of Foz do Iguaçu, during the Rabies vaccination senses in August of 2014. The survey for cVL in FI was carried out from 06–22 October 2014.

The city of Santa Terezinha de Itaipu (STI, 25° 21' 44" S, 54° 29' 17" W) is located 218–312 m above sea level. It has an area of 248,133 Km^2^, of which 96.0% are considered rural, 3.4% urban and 0.6% lake and forest. About 22,783 habitants live there, 90.38% of which live in urban areas [20]. The total dogs’ number was 5,696 (data obtained from Sanitaire Vigilance). For this survey dogs was sampled in November 2015.

Two Transects (T1 and T2) were drawn between Foz do Iguaçu and Santa Terezinha de Itaipu, comprising only rural and forest areas. T1 ran from Foz do Iguaçu to the natural reserve of Iguaçu National Park, whereas T2 ran from this park to STI. In each 0.86 km five dogs from residences were surveyed from 03 to 21 of November 2014.

### Dogs sampling

**Fig 1:**
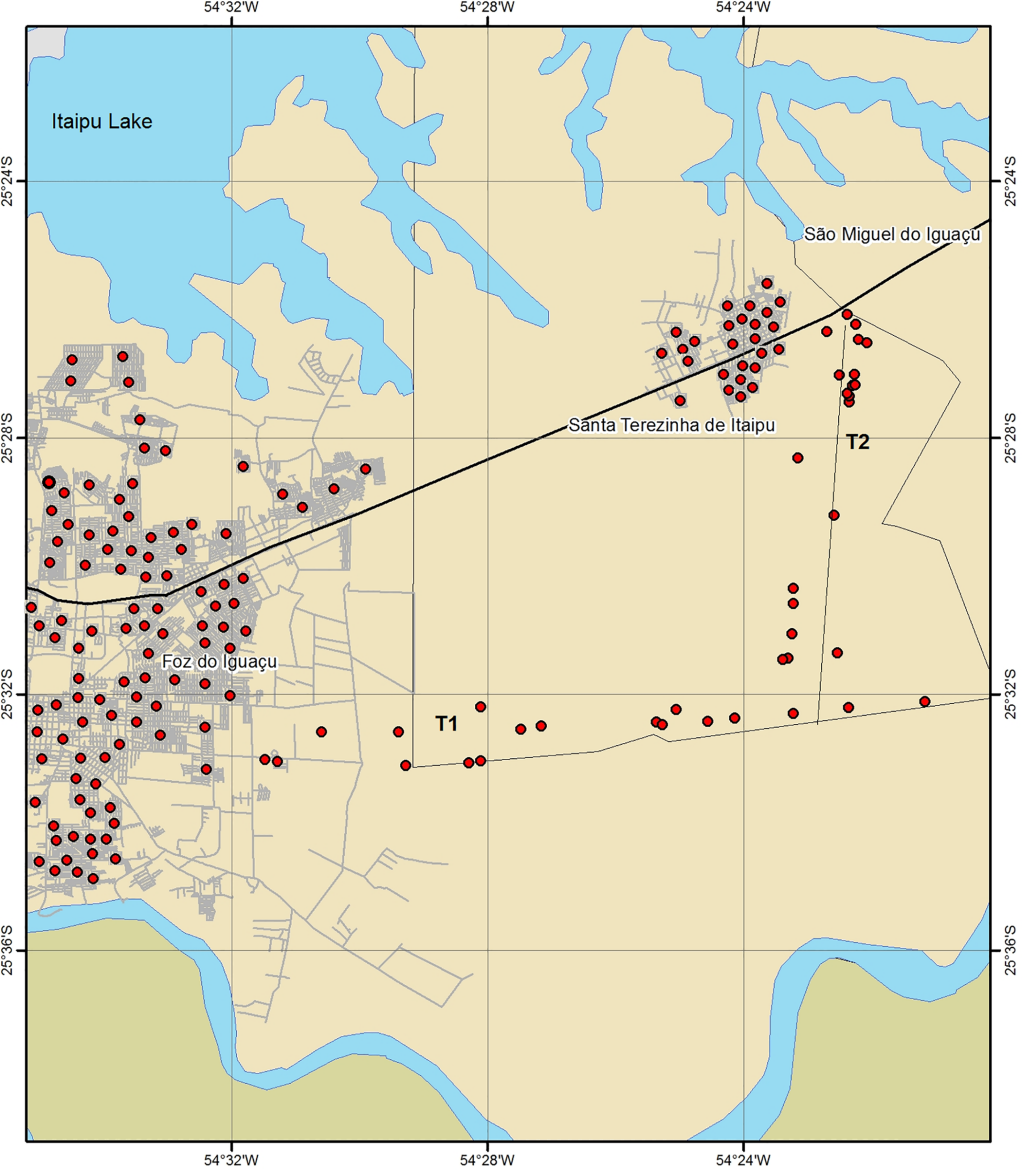
In the 196 sites (pointed in the maps) from three areas of the extreme -west of the Southern of Brasil (Foz do Iguaçu, Santa Terezinha de Itaipu and transect between the two cities) dogs were sampled to determine the seroprevalence to leishmaniases. In each site blood of five (or more) dogs were sampled and examinated serologically according to Brazil Health Ministery recommendation. The two test (DPP and Immunoassay) were realized simultanealy. We considered a positive animal when two serological exams were positives.

The urban areas (FI and STI) were divided in 526 patches of 400 m^2^ according to [21, 22]. Due to the limitation of number of CDC-LT traps, 123 patches from FI, 40 from the transects (in each 0.86 km) and 33 from STI were selected due to be the most appropriated for the proliferation of cVL according to the ‘worst scenario’ criterion [22, 23]. The patches (Fig 1) were geo-referenced using GPS equipment and the ArcGIS 10.1 software [24].

Five dogs from each patch (usually a house) have their blood sampled (irrespective of their clinical status). When five dogs were not available in this house, dogs from the neighbourhood were included, up to five. All sampled dogs were owned, and no stray dogs were collected. First, the dogs were examined for clinical signs of the disease and each of them was assigned an individual data file that included its identification, traits, behaviour, migration history and health-related issues. Each file included the following information: breed, gender, age, size, night resting place, and weather the dog was on a leash during the night or not, migration history, use and periodicity of repellent, clinical signs of leishmaniasis, and nutritional state. The presence of one or more of the following signs was taken as a clinical indication of cVL: lymphadenopathy, onychogryphosis, cutaneous lesions, weight loss, conjunctivitis and alopecia. In the second step, blood samples of the dogs were collected by venepuncture of the jugular or the cephalic using a disposable syringe and needle (25x7), transferred into 10 mL polypropylene tubes, and processed 3–4 h after collection. In the laboratory blood was centrifuged at 1000 x*g* for 5 min and sera were separated and stored at −20°C until analysed by two serological methods. Finally the extrinsic factors (environmental: vegetal cover, cement, bare soil cover + covered surface with unused materials, number of chickens, repellent, number of *Lu*. *longipalpis* and presence of infected dogs in the neighbourhood) (Table 1) were recorded.

**Table 1:** Extrinsic and intrinsic variable assessed in this study.

Group	Variable Recorded	Explanation
**Extrinsic**	Site	Foz do Iguaçu, transects and Santa Terezinha de Itaipu
	Vegetal cover	Number of fruit trees of the household Percentage of vegetal cover of the patch
	Cement	Percentage of cement of the patch
	Bare soil cover + Covered surface with unused materials(intermedium)	Percentage of soil and surface unused of the patch
	Number of chicken	Number of chicken
	Repellent	Use of repellent
	Number of *Lu*. *longipalpis*	Number of *Lu*. *longipalpis*
	Infected neighbor dog	Presence of infected dogs in the neighborhood
**Intrinsic**	Age	Age of the dog
	Ambulate	Dog that moves from different places
	Autochthonous	Dog from the site
	Allochthonous	Dogs from other places
	Sleep outside	Dogs that sleeps outside a house
	Size	Size of the dog
	Nutrition	Nutrition state of the dog
	Ticks	Presence of Ticks
	Fleas	Presence of Fleas

All procedures involving dogs were conducted in strict accordance with the regulations outlined by the National Council for the Control of Animal Experimentation (CONCEA), and all efforts were made to minimise suffering.

The Animal Care and ethical Committee of the Federal University of Paraná, under protocol no. 044/2014.The owners received the results of the examination, along with general prevention recommendations based on reducing effective vector-reservoir/vector-human contact.

### Serologial procedures and molecular *Leishmania* identification

Two serological tests were used in the serological survey: an immunoassay test (using crude antigen cELISA), as proposed by Mazieiro et al., 2014 [10], and the Dual Path Platform (DPP® cVL, Biomanguinhos).

As recommended by the Brazilian Ministry of Health, only dogs that tested seropositive in both tests were considered infected [25,26]. From these animals, popliteal lymph nodes were taken using a disposable syringe and needle (1.2 x 40 mm) and test for the presence of *Leishmania* parasites using Polymerase Chain Reaction (PCR). DNA extraction was performed using the Wizard^®^ Genomic DNA purification kit in accordance with the manufacturer’s recommendations. The DNA pellet was dissolved in 50 μL of Tris-EDTA buffer incubated in water bath at 65°C for 30 min, and stored at −20°C until analysis. PCR was performed with internal transcribed spacer (ITS) primers followed by RFLP as described by Schonian et al. 2003 [27] for *Leishmania* identification. The Cytochrome B1/B2 was used as an internal control to verify DNA amplification [28]. Positive PCR products (lymph nodes samples) with pattern of RFLP electrophoresis different from the reference *Leishmania* (i.e. the most common *Leishmania* species in the Brazil: *L*. *infantum*, *L*. *braziliensis*, *L*. *amazonensis*) were sequenced to confirm the identification. The sequencing was commercially performed by the Macrogen Inc. (Seoul, South Korea). The resulting sequences were deposited in the Genbank with the following accession numbers MF945579 to MF945584. The sequences were aligned using the MAFFT 7.0 [29] in the Guidance web server [30–32]. The final alignment was composed of 255 bp sequences, including the indel mutations. The identification was done through a Neighbor-Joining (NJ) tree constructed in MEGA 7.0 [33], using the substitution model (K80 –[34]) defined by the software jModeltest 2.1.10 [35], and the robustness of the NJ tree was assessed using 1,000 bootstrap replicates.

### Statistical analyses

Each positive patch was recorded and transformed for input into the Geographic Information System (GIS) environment program. After the data were reclassified, they were converted to the raster format, to enable map algebra, highlighting the sites with the greatest number of cases [24]. After the map algebra was completed, the data were converted to vector format using cross-tabulation.

To assess the clinical diagnostics of cLV, differences in clinical signals between seropositive and seronegative groups, recorded in the epidemiological questionnaire, were tested using the chi-square (for FI: without clinical signals, with clinical signals, state mind active, weight loss, adenomegaly, alopecia, skin lesions, mucosae lesions, hiperkeratosis, muscular atrophy; for T1+T2: without clinical signals, with clinical signals; all together: without clinical signals, with clinical signals, state mind active and apathic, weight loss, adenomegaly, alopecia, skin lesions, mucosae lesions, hiperkeratosis, muscular atrophy) or Fisher's exact test (F for FI: lethargic; for T1+T2: eye injury; all sites: lethargic) in the R 3.3.3 [36]. The odds ratios (OR), with a confidence interval (CI) of 95%, was employed to measure the association between each clinical signal and the groups (seronegative and seropositive dogs).

The risk analysis started with pairwise correlations between infection and the 18 variables recorded: residence, % of trees, % of cement coverage, infected neighbor dogs, sleeps outside, presence of chickens, number of chickens, presence and abundance of *Lutzomyia longipalpis*, size of the animal, age, dog coming from the same neighborhood (autochthonous) or from other places (allochthonous), use of repellent, nutritional status, presence of ectoparasites. The variables with significant correlation were then tested in glm (generalized linear model) using a binary logistic regression. Odds Ratio (OR) and its 95% confidence Interval were calculated [37].

Moreover, to assess the direct cross influence among all variables, including the infection, two Path Analyses was performed using the package ‘plspm’ 0.4.7 [38] in R 3.3.3. First, an analysis using only the environmental variables (see Table 1) was performed to test their effects on the proportion of infected dogs at each patch. This analysis assessed the factor that provided opportunities for a dog to become infected by the parasite. Second, a Path Analysis was carried out using only the intrinsic characteristics of the dogs (see Table 1) to test the risk factors that predisposed dogs to get infected with *L*. *infantum*. This analysis tested which factors affect the dogs’ likelihood of becoming infected by the parasite. In both analyses, the abundance data were logarithmized and the critical p values were corrected using the B-Y method [39].

## Results

### cVL seroprevalence in the extreme-west of Paraná state

In the three areas a total of 196 patches were surveyed: 123 in FI, 33 in STI and 40 in the transects (T1 + T2). The number of patches with seropositive dogs were 67 (54.4%), 16 (48.5%) and 9 (27.3%), respectively. In the rural areas fewer dogs were positive for the infection. In fact, infection rates were always higher at urban sites (Table 2).

**Table 2:** Number of positive patches to cVL (before the bar), number of total patches (after the bar) and percentage of positive patches to cVL from three strata from Foz do Iguaçu, Santa Terezinha de Itaipu and the transects between two cities. **—**No urban area; * small community.

Area	Foz do Iguaçu	Transects	Santa Terezinha de Itaipu	TOTAL
**Urban**	53/93	-	08/29	61/122
	(56.9)	-	(27.6)	(50.0)
**Peri-urban**	13/27	06/40	1/4	20/71
	(48.1)	(15.0)*	(25.0)	(28.2)
**Rural**	1/3	10/40	-	11/43
	(33.4)	(25.0)	-	(25.6)
**TOTAL**	67/123	16/40	09/33	92/196
	(54.4)	(25.0)	(27.3)	(47.0)

Among the 1,129 dogs sampled, 785 (69.5%) did not presented clinical signals and 344 (30.5%) presented clinical signals. The percentage of seropositive dogs was 23.8% (185 of a total of 777 dogs) in FI, 9.1% (16 of a total of 176) in the transects, and 5.1% (9 of a total of 176) in STI (Table 3).

**Table 3:** Dogs sampled (N), seropositive dogs to cVL (N+) and its percentage in three sites in the extreme-west of the Paraná state, Southern of Brazil: The Foz do Iguaçu (FI) city was devised in four areas (A, B, C and D), Santa Terezinha do Itaipu (STI) in two areas (A = north and B south), and two transects (T1 and T2) between the two cities (FI and STI). A total of 1129 dogs were sampled.

Foz do Iguaçu			Transects			Santa Terezinha de Itaipu		
Area	N	N+	Area	N	N+	Area	N	N+
**A**	170	48	T1	90	11	A	60	4
		(28.2)			(13.0)			(7.1)
**B**	157	44	T2	86	5	B	110	5
		(28.0)			(5.9)			(3.7)
**C**	273	44						
		(16.1)						
**D**	177	49						
		(27.6)						
**Total**	777	185		176	16		176	9
		(23.8)			(9.1)			(4.7)

### Clinical signs of the cVL

Of the 21 clinical signs surveyed that were compatible with visceral leishmaniasis, 11 were statistically significantly different between seropositive and seronegative groups: State mind (active, apatic and lethargic) weight loss, adenomegaly, alopecia, skin lesions, mucosae lesions, hyperkeratosis, onychogryphosis, muscular atrophy, eye injury, gastrointestinal disorder. The majority of dogs presented more than one clinical signal (Table 4).

**Table 4:** Clinical classification of seropositive dogs in three regions in the extreme west of Paraná state, Southern Brazil. OR: Odds Ratio.–Non observed date. * seropositive dogs in ELISA and DPP tests /total of dogs with clinical signals.

Clinical classification	Foz do Iguaçu			Transects			Santa Terezinha de Itaipu			Total		
	%	OR	p value	%	OR	p value	%	OR	p value	%	OR	p value
	positive			positive			positive			positive		
	/total*			/total*			/total*			/total*		
**Without signals**	11.7%	0.31	0.000	0.9%	0.09	0.020	4.9%	1.71	1.000	8.9%	0.32	0.000
	(62/528)	(0.21–0.45)		(1/115)	(0.01–0.86)		(7/142)	(0.20–14.93)		(70/785)	(0.22–0.45)	
**With signals**	29.7%	3.17	0.000	8.2%	10.17	0.020	2.9%	0.58	1.000	23.7%	3.09	0.000
	(74/249)	(2.17–4.64)		(5/61)	(1.16–89.21)		(1/34)	(0.06–4.91)		(80/344)	(2.18–4.39)	
**State mind**												
**Active**	16.6%	9.79	0.003	3.0%	0.24	0.273	4.0%	0.08	0.130	12.5%	0.30	0.000
	(123/742)	(0.16–0.68)		(5/167)	(0.02–2.36)		(7/173)	(0.00–1.04)		(135/1082)	(0.16–0.57)	
**Apatic**	18.2%	1.18	0.766	11.1%	4.05	0.273	33.3%	11.85%	0.130	26.3%	2.42	0.030
	(4/22)	(0.39–3.56)		(1/9)	(0.42–38.86)		(1/3)	(0.95–146.89)		(10/38)	(1.15–5.10)	
**Lethargic**	55.6%	6.07	0.010	-	-	-	-	-	-	55.6%	8.40	0.003
	(5/9)	(1.61–22.94)		-	-					(5/9)	(2.23–31.66)	
**Weight loss**	36.7%	3.00	0.000	0	0.00	1.000	0	0	1.000	28.6%	2.83	0.000
	(18/49)	(1.62–5.54)								(18/63)	(1.56–5.03)	
**Adenomegaly**	45.2%	5.44	0.000	4.6%	1.41	0.556	0	0	1.000	37.0%	5.12	0.000
	(46/101)	(3.47–8.54)		(1/22)	(0.15–12.74)					(47/127)	(3.38–7.75)	
**Alopecia**	33.7%	2.83	0.001	10.00%	4.22	0.139	0	0	1.000	25.7%	2.64	0.000
	(33/98)	(1.77–4.53)		(2/20)	(0.72–24.69)					(35/136)	(1.71–4.07)	
**Skin lesions**	36.2%	2.90	0.001	3.5%	1.01	1.000	0	0	1.000	23.1%	2.08	0.014
	(17/47)	(1.55–5.44)		(1/29)	(0.11–9.01)					(18/78)	(1.19–3.64)	
**Mucosae lesions**	36.8%	2.95	0.003	3.4%	1.01	1.000	0	0	1.000	35.0%	3.77	0.000
	(14/38)	(1.48–5.86)		(1/29)	(0.11–9.01)					(14/40)	(1.92–7.40)	
**Hyperkeratosis**	35.7%	2.74	0.019	0	0.00	1.000	0	0	1.000	32.3%	3.25	0.005
	(10/28)	(1.23–6.09)								(10/31)	(1.50–7.06)	
**Onychogryphosis**	23.3%	1.65	0.302	0	0.00	1.000	0	0	1.000	14.6%	1.28	0.496
	(7/30)	(0.69–3.95)								(7/48)	(0.56–2.92)	
**Muscular atrophy**	60.0%	7.35	0.003	0	0.00	1.000	-	-	-	50.0%	6.75	0.002
	(6/10)	(2.04–26.41)								(6/12)	(2.15–21.23)	
**Eye injury**	12.9%	0.77	0.804	20.0%	10.1	0.039	6.2%	1.45	0.541	(7/57)	1.04	0.834
	(4/31)	(0.26–2.26)		(2/10)	(1.60–63.74)		(1/16)	(0.16–12.65)			(0.46–2.35)	
**Gastrointestinal disorder**	50.0%	5.33	0.292	0	0.00	1.000	0	0	1.000	20.0%	1.86	0.468
	(1/2)	(0.33–86.01)								(1/5)	(0.20–16.81)	

### Parasite identification

**Fig 2:**
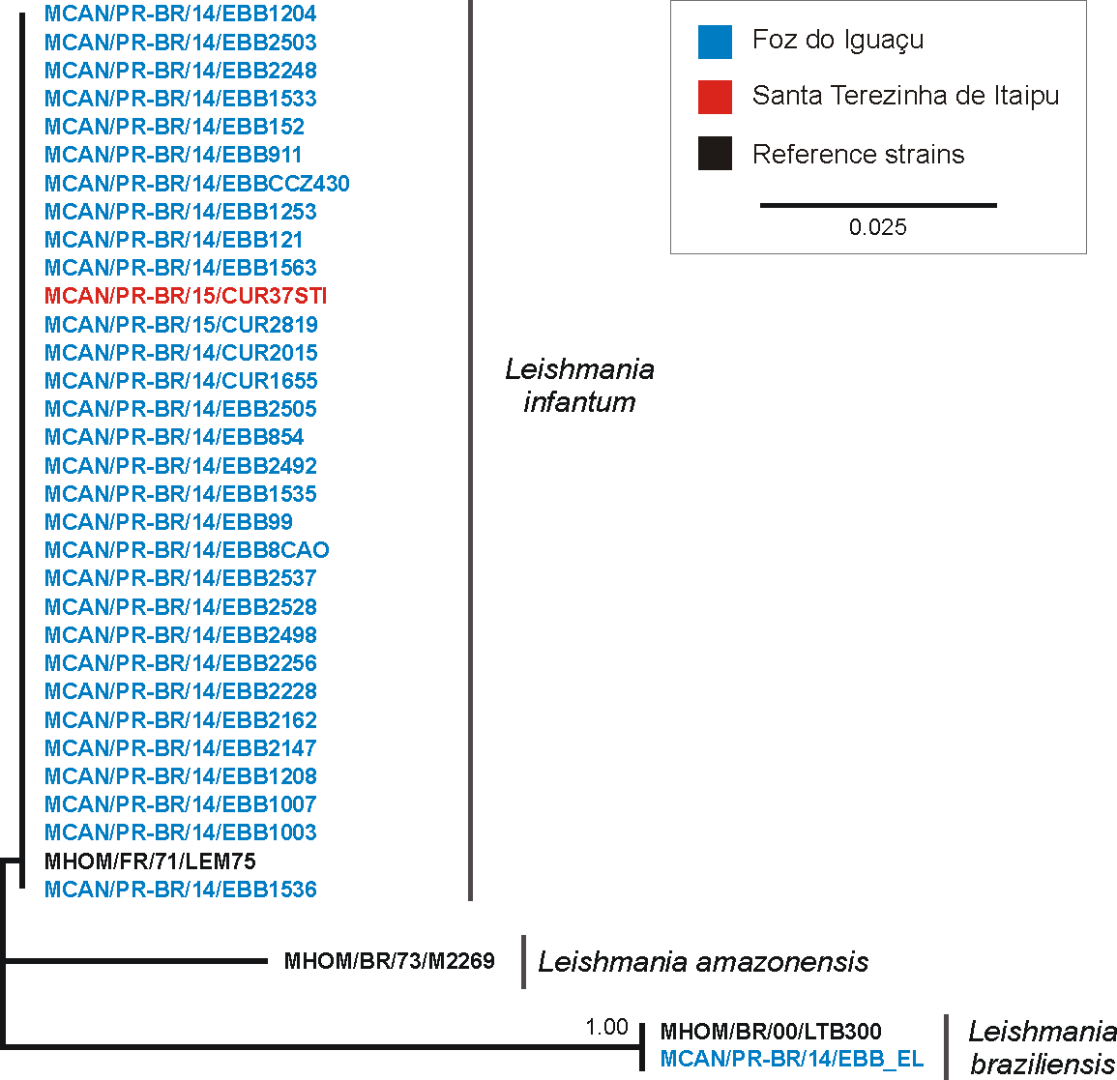
Neighbor-Joining tree of ITS1 of *Leishmania* individuals isolated from dogs from the extreme west of the state of Paraná, Brazil.

In Foz do Iguaçu, of the 124 dogs that tested positive in the two serological tests, the PCR of lymph node samples were also positive in 111 (89.8%). The PCR-RFLP test present bands consistent with the pattern produced by the *L*. *infantum* reference strain in 110 dogs, and the NJ tree supported their identification. One dog from Foz do Iguaçu presented RFLP pattern similar to *L*. *braziliensis*, and the ITS1 NJ tree supported its identification (Fig 2). Of the 37 seronegative dogs in both serological test and with clinical signs of cVL, 14 (37.8%) were positive to *L*. *infantum* by PCR-RFLP.

In the transect areas, 16 seropositive dogs were tested using PCR-RFLP and samples from six (37.5%) dogs yielded bands compatible with *Leishmania* spp. The PCR-RFLP showed that five of these dogs presented patterns similar to *L*. *infantum*, all of which were allochthonous (dogs coming from FI). In one dog autochthonous its PCR-RFLP pattern was consistent with *L*. *braziliensis* (the parasite of this dog was not sequenced).

In STI, of the nine dogs that were positive for both serological tests, seven yielded a PCR-RFLP pattern consistent with *L*. *infantum*, and its identification were corroborated by the NJ tree. However, among the seven dogs that tested positive by PCR-RFLP, five were from FI, and only two were authoctonous.

### The spatial distribution of the seropositive dogs

**Fig 3:**
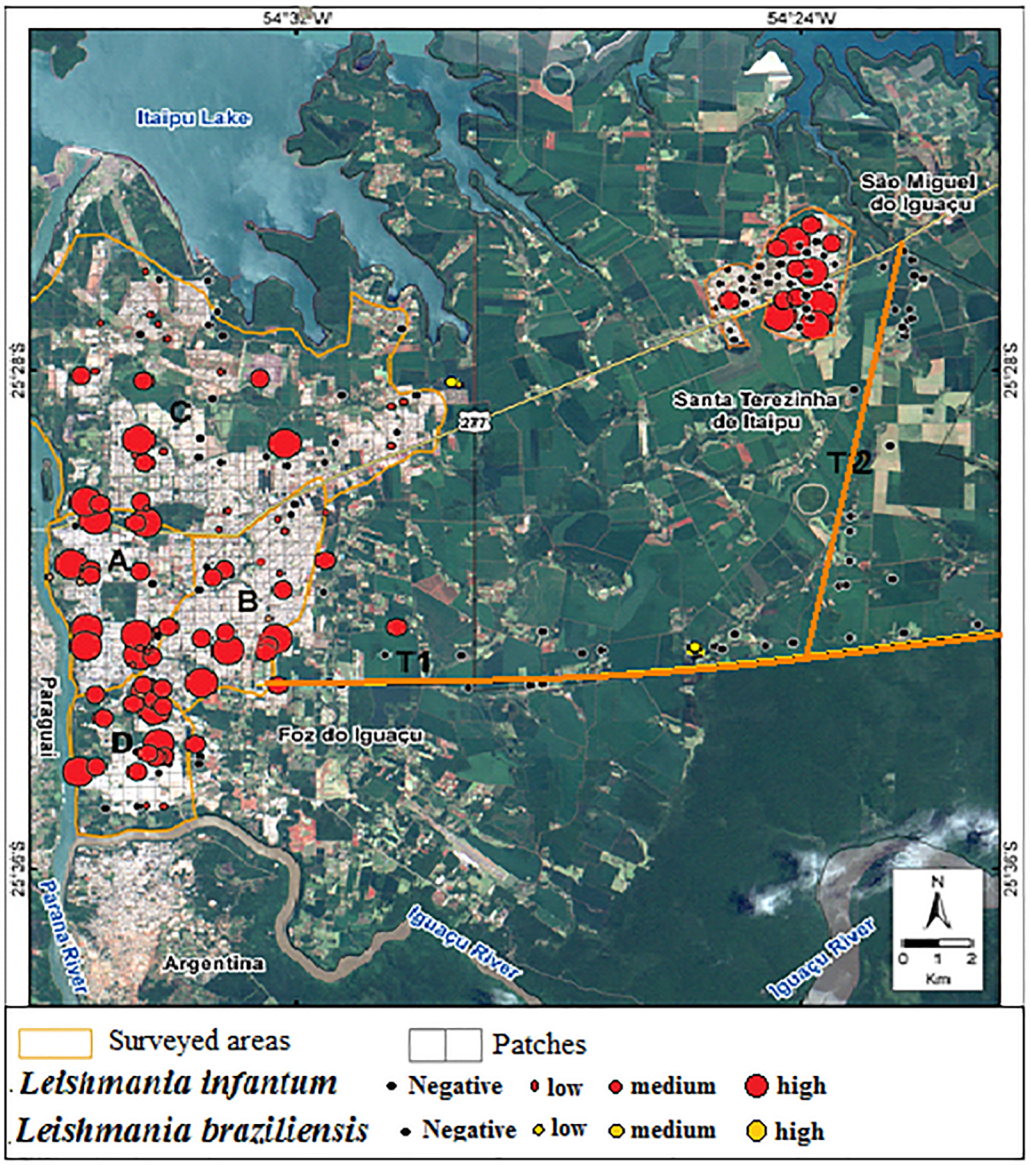
Patches sampled and spatial distribution of *Leishmania* spp. in the extreme west of Parana state, Southern Brasil. The higher prevalence were observed in area A and D.

In FI, the highest prevalence of cVL was in areas A and D (Table 3, Fig 3). In area B, seropositive dogs were present on both sides of BR-277 (largest Brazilian road in this region). In STI, seropositive dogs were found in the two studied areas (right and left side of BR-277 road). In the transects, the dogs that tested seropositive for *L*. *infantum* originated from FI. The dogs that tested seropositive for *L*. *braziliensis* were observed near the Iguaçu National Park.

### Risk analyses

The glm analysis supported that percentage of trees, percentage of cement, infected neighbour, animal size, nutritional status, outside sleeping, presence of ectoparasites and abundance of *Lu*. *longipalpis* significantly affected dogs’s infection rates by cVL in FI. In STI, the presence of infected dogs in the neighbourhood influenced the rate of cVL infection the most. In the transects, the behaviour (living on the street) and the presence of infected dogs in the neighbourhood influenced dogs’ infection rates the most. When all animals at all sites were evaluated, eight variables were significant (Table 5).

**Table 5:** Intrinsic and extrinsic variables that showed significance to canine visceral leishmaniasis. NS: non significant values.

Variables	Foz do Iguacu		Transects		Santa Terezinha de Itaipu		Total	
	OR	p-value	OR	p-value	OR	p-value	OR	p-value
	(95% CI)		(95% CI)		(95% CI)		(95% CI)	
**Trees**	0.99	0.041	NS		NS		0.99	0.033
	(0.98–0.99)						(0.98–0.99)	
**Infected neighbours**								
**no**	Reference		Reference		Reference		Reference	
**yes**	3.15	0.000	3.36	0.011	7.36	0.000	3.46	0.000
	(2.09–4.82)		(1.33–8.78)		(2.88–22.75)		(2.45–4.94)	
***Lu*. *longipalpis***								
**no**	Reference						Reference	
**yes**	1.86	0.001	NS		NS		1.79	0.001
	(1.25–2.75)						(1.25–2.55)	
**Size**								
**small**	Reference						Reference	
**medium**	1.72	0.014	NS		NS		1.63	0.008
	(1.11–2.65)						(1.13–2.34)	
**big**	2.67	0.000	NS		NS		1.97	0.002
	(1.58–4.47)						(1.26–3.04)	
**Nutrition**								
**good**	Reference						Reference	
**regular**	NS		NS		NS		1.96	0.011
							(1.15–3.28)	
**bad**	4.20	0.008	NS		NS		2.88	0.037
	(1.40–12.18)						(1.01–7.67)	
**Fleas**								
**no**	Reference						Reference	
**yes**	1.78	0.004	NS		NS		1.51	0.017
	(1.19–2.66)						(1.07–2.11)	

**Fig 4:**
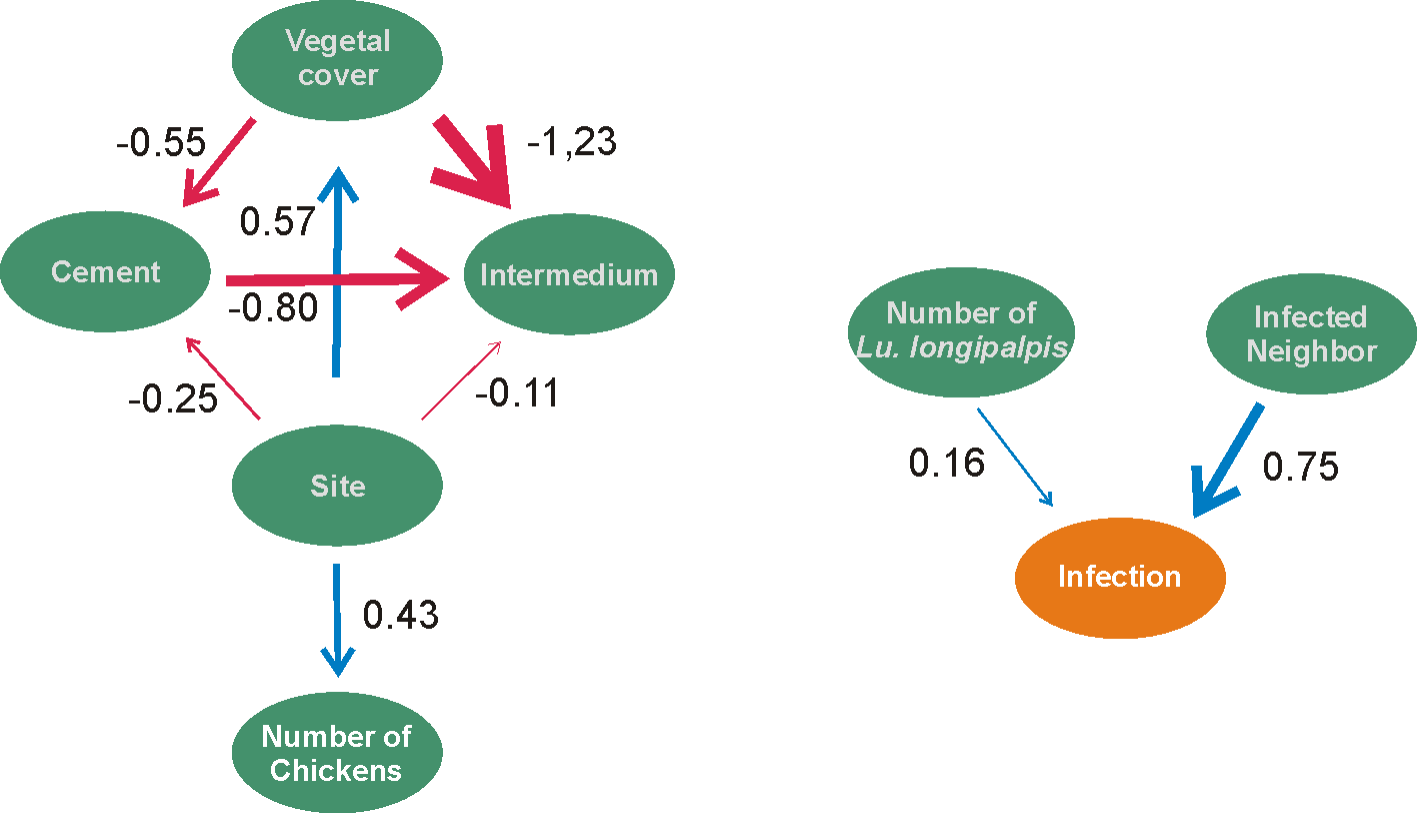
Path analysis with extrinsic (environmental) characteristics that affect the infection rate in dogs from western region of the Paraná State, Brazil. Blue arrows represent positive effect, and red arrows represent negative effects.

**Fig 5:**
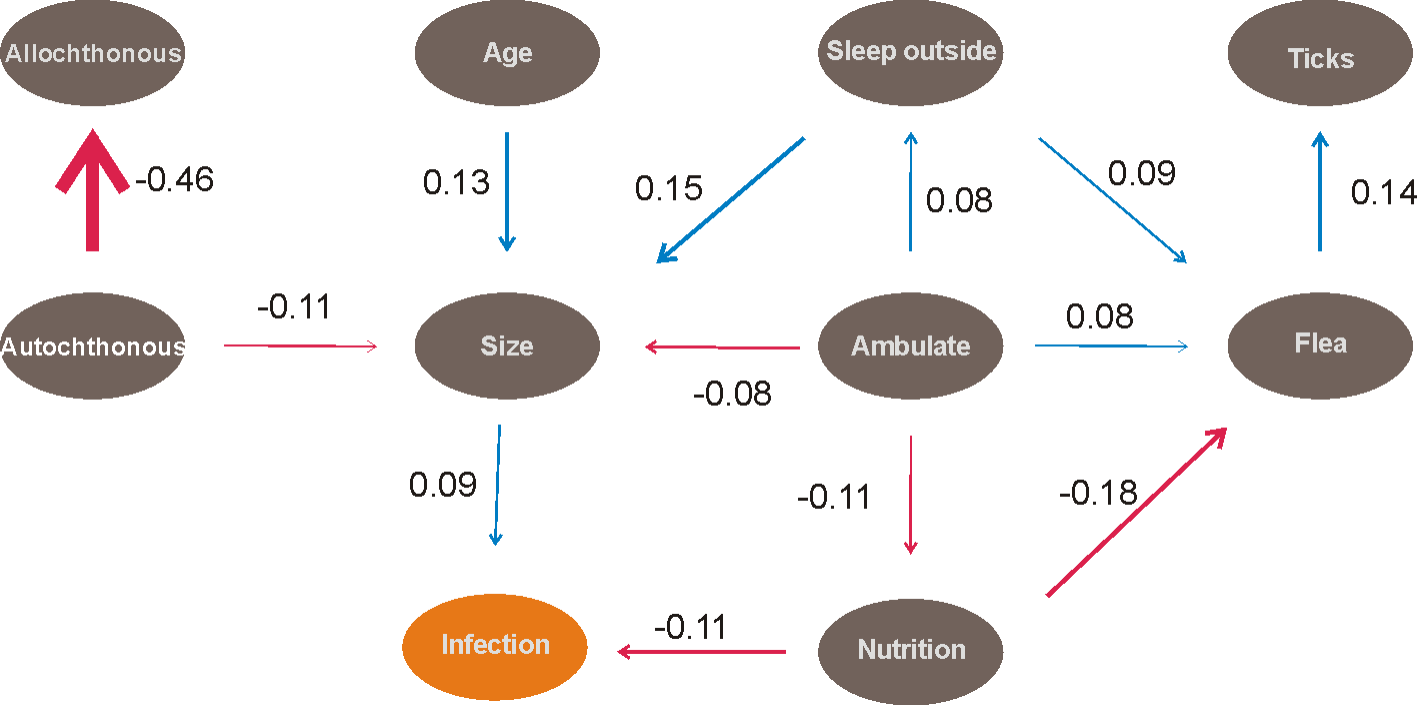
Path analysis with intrinsic characteristics of the dogs that affect their probability of infection in the western region of the Paraná State, Brazil. Blue arrows represent positive effect, and red arrows represent negative effects.

The Path Analysis of extrinsic (environmental) factors supported that the presence of infected dogs in the neighborhood (path coefficient of 0.75, p < 0.01) and the abundance of *Lu*. *longipalpis* (path coefficient of 0.16, p < 0.01) had a positive influence on the proportion of infected dogs at each patch (R^2^ of the model = 60%) (Fig 4). The Path Analysis of intrinsic dog characteristics supported that dog size was positively correlated with the probability of a dog becoming infected with *Leishmania* spp. (path coefficient of 0.09, p < 0.01) and nutrition was negatively correlated with it (better nutrition less risk and vice versa) (path coefficient of -0.11, p < 0.01. (R^2^ of the model = 4%) (Fig 5).

## Discussion

In the three far-west sites surveyed in the state of Paraná, 210/1,129 (18.6%), dogs were seropositive for *Leishmania* infection. The highest prevalence was observed in FI (23.8%), especially in areas A, D and B (with approximately 28% of the dogs infected). In area C, the prevalence rate was lower (16.13%). But, seropositive dogs were still present in the patches sampled (26.2% of the patches), indicating that the introduction of cVL in this area is more recent. Several authors have suggested that, in the new and old worlds, the prevalence of cVL varies according to the geographical region, parasite pressure and diagnostic method. For example, in the old word the rates of infection ranged from 5% to 80% in Italy, Spain, Portugal and France, averaging 23.3%. However, there are sites in the first three countries where there is a prevalence of 80%, while the greatest total prevalence rate of 43% was found in France [40–42].

The regional prevalence rates of cVL in Brazil also vary in the local and regional scales: from 6.7 to 29.3% in the Northeast; from 47.8 to 59.3% in the North; from 10 to 45.2% in the Southeast; and from 9.3 to 65% in the Center-West. In the South (where LV is more recent) a survey in Rio Grande do Sul (2009 to 2010), conducted in 34 municipalities and using 5,430 dogs, found a prevalence rate of 20.8% [4, 7, 43–48]. In the neighboring countries around our study area, Argentina and Paraguay, cVL has a prevalence of 7.2 to 16.8 and 3.1 to 11.8% respectively. However, in those countries, only the rk39 test (lateral-flow assay) was used. The sensitivity and specificity of this test is not consistent with the recommendations of Brazil’s Health Ministry [21, 22, 49]. According Quinnell et al., [50], the sensitivity and specificity of the rk39 varied across studies, but their combined sensitivity to detect the clinical disease was 86.7% and to detect infection was 59.3%. Grimaldi et al., [51] showed that the sensitivity of the TRDPP®, one of the methods used in our study, was 98% (59/60 samples) for the detection of dogs with with disease, but lower (47%) in identifying parasite-positive dogs without signs of cVL. In the extreme west of the state of Paraná, among the 1,129 dogs sampled, 28.1% presented clinical signs compatible with cVL, and 50.8% of them were seropositive. On the other hand, even dogs without clinical signals were positive for serological tests (8.1%). Several studies have shown that the prevalence of clinical signs is between 3 to 10% [4,52–55]. However, we found an alarmingly greater prevalence (about 30%). There are other diseases with clinical signs like cVL, including ehrlichiosis, dermatophytosis and canine scabies. For this reason, fast and better diagnoses are essential to understand the epidemiology, to control VL, and to confirm all seropositive cases of cVL. In addition, to determine the magnitude of the infection, we employed a PCR-RFLP approach to complement the diagnoses.

In general, molecular techniques are efficient to detect infections by *L*. *infantum*, even in the early stages, while serological techniques are more efficient at the advanced stages, when IgG production is established and can be more easily detected. In a longitudinal study, Quinnell et al. [56] reported a 98% sensitivity of the PCR in parasite-positive samples in the initial stage, but this decreased to 68% in the chronic phase. In our study, using lymph nodes samples the PCR technique supported that 37.8% of the dogs with clinical signals, but seronegative, were infected by *L*. *infantum*. It supports the necessity of working with different techniques to complete the diagnosis and reduce the infected number of animals. The RFLPs technique with ITS marker, showed more bands that suggested in the original article [27] in some isolates, and they were sequenced, confirming *L*. *infantum* as principal specie (Fig 2). Alternatively, *L*. *braziliensis* parasites were found in an autochthonous dog in the transect areas and other in Foz do Iguaçu. This is the first record of *L*. *braziliensis* in dogs from this region. This supports that both *L*. *infantum* and *L*. *braziliensis* are sympatrically and perhaps synthopically distributed in this region, especially in peri-urban areas.

In the Brazilian side of the triple border, the widespread distribution of seropositive dogs, the abundance of the vector *Lu*. *longipalpis* and the high percentage of older dogs infected throughout FI suggest that the parasite cycle can be established in this region, and that cVL may be considered endemic to this area. Moreover, authocthonous cVL in STI cases suggests that Foz do Iguaçu is a gateway (BR-277) for cVL in the western portion of the state of Paraná. Furthermore, the presence of infected dogs in the transects, although originated allochthonous, supported that this road is a possible dispersion route for the disease between these cities. Besides this rural road in the transects, the highway BR-277, connects both cities and runs to the city of Paranaguá, in the extreme east of the state of Paraná, is likely an important route of dispersion of infected dogs, and consequently the parasite and the disease, to other regions of the state.

The geo-referencing data of seropositive dogs showed that the sectors with high rates of canine positivity and the highest canine infection rates were associated with forest fragments and streams (see Fig 3). As part of the urbanisation of Foz do Iguaçu, Atlantic Forest vegetation was preserved in fragments in the urban area, especially in areas A and D which, in turn, support the phytophysionomic characteristics of semideciduous forests. Similarly, from the 1980s, with the development of the city that emerged in conjunction with the construction of the Itaipu hydroelectric power plant, and now, with the Municipal Plan for Urban Forestation (in its final steps), there has been a tendency to combine vegetated areas with buildings and urban structures. Thus, area C is currently booming with the construction of residential condominiums. Moreover, more recently an avenue linking areas A and B was built, passing through a forest reserve. The relationship between forest and urban occupation is a historical feature and was socially constructed by the population of Foz do Iguaçu. In particular, the maintenance of green areas in the urban portion is a capital of the city. The green areas can maintain the cycle of *Lu*. *longipalpis* and make it difficult to control cVL.

The search for risk variables for the occurrence of cVL has shown that both intrinsic and extrinsic variables are important in the maintenance of the endemism of this disease. Our Path Analysis of the extrinsic variables supported that the abundance of *Lu*. *longipalpis* and the presence of infected dogs in the neighbourhood are the variables that affect the presence of infected dogs. Infected reservoirs are a source of parasites, and while *Lu*. *longipalpis* facilitates the transmission of the parasite between dogs. Vertical transmission of the disease is also possible, e.g. during pregnancy [57]. In this way, the proximity of infected dogs and the abundance of *Lu*. *longipalpis* provide the opportunity for *L*. *infantum* to parasitize a non-infected dog.

On the other hand, a factor that affects the capacity of dogs to avoid the infection is their nutritional status and size. Large dogs have larger areas to attract the vector and to be bitten by the infected Phlebotominae. Malnourished dogs have weaker immune response, which is further compromised by the cVL infection. Moreover, other factors, especially dogs’ capacity to migrate, have a greater importance on the dispersion of VL to different regions. Similarly, the presence of fleas increases the infection rate (1.51 in all regions and 1.78 in dogs from FI). Fleas are known to transmit diseases between individuals see Mencke 2013 [58]. Specifically, in recent years, Dantas-Torres (2011 [59]) and Paz (2013 [60]) postulated that fleas and ticks may act as *Leishmania* vectors. In this way, the influence of the presence of fleas on the infection of cVL may be resulted from direct transmission of *Leishmania* by the insect, or resulted from the decrease in the health of the dog by the bite of fleas or transmission of other diseases. Thus, once sand flies have been registered in the three areas surveyed, fleas could amplify the prevalence of the cVL.

Alternatively, our results did not support that chickens affect the probability of infection of dogs by *Leishmania*. Some studies supported that the blood of chickens are most preferred food supply by *Lu*. *longipalpis*, followed by blood of dogs [61,62]. Furthermore, chickens may be important to the maintenance of sand fly populations, and amplify the leishmaniasis prevalence [63]. However, although about 21% of the patches in FI presented chickens, 62% in the transects and 46% in STI, our results does not supported this hypothesis. Thus, the dogs, as a food supply, may be enough to maintain the cycles of the vector and the parasite.

In endemic areas, cVL represents only a small part of the problem [64], because the disease is complex, with several variables involved as such environmental, socio-cultural and geopolitics. VL is a zoonosis, and the domestic dog participates in the biological and epidemiological cycles. In a social survey conducted in the city of Foz do Iguaçu, epidemiological records confirm that the average number of dogs per household was 1.35, while the number of people per household was 4.0. In developing countries, companion animals currently play a central role in families living in large cities, and this tendency has been observed in Brazil. With the decreasing number of individuals per household, dogs and cats have become more important to help people with mental disorders, and other disabilities [65]. Thus, more reliable diagnostic approaches and better drugs for treatment are essential to identify seropositive dogs and dogs that are parasitized, since isolating them, or treating them as soon as possible, would reduce parasite pressure, reducing the risk for the human population and sparing the rest of the canine population. In the example studied here, controlling the disease in the infected dogs would spare the other, healthy dogs (75% of the population). However, if the disease is not controlled, the continuous infection of vectors and hosts will allow for the propagation of LV. Therefore, it is urgent that researchers in areas that are considered endemic for visceral leishmaniasis discuss this issue in international forums specific to *Leishmania* and leishmaniasis and seek a global consensus to avoid the unnecessary suffering of people and animals.

The increased prevalence of cVL poses a public health problem that needs to be dealt with. This includes the decision to treat or not to treat infected animals. If treatment is the choice of action, who will pay for it in developing countries? Which organizations will monitor the treatments, and how to organize a public veterinary service for the care of dogs that belong to low-income people? All those issues need to be discussed. For over 10,000 years the dog has been considered man's best friend. Therefore, it would seem inconceivable for society to kill its best friend. Avoiding the unnecessary death of animals is one of the most popular goals of today’s affluent Brazilian society.

## Conclusions

Our results support that cVL is endemic in the extreme west of the state of Paraná, in areas bordering Argentina and Paraguay. For this reason, border surveillance systems are the key to avoiding this silent disease. The high prevalence of cVL in dogs (23.8%) and the widespread dispersion in FI and the next city (STI) indicates that cVL is endemic to the west area. It is also possible to estimate that in FI approximately 13,085 dogs may be infected with *L*. *infantum* out of a total of 54,983. This suggests a worrying scenario to the establishment of human visceral leishmaniasis and the spread of the disease by dogs to other areas of this region.
